# Uhthoff`s phenomenon 125 years later - what do we know today?

**Published:** 2016

**Authors:** JA Opara, W Brola, AA Wylegala, E Wylegala

**Affiliations:** * Academy of Physical Education in Katowice, Polands; ** District Hospital in Konskie, Poland; *** Ophthalmology Clinic, Medical University of Silesia in Katowice, Poland

**Keywords:** hyperthermia, Multiple Sclerosis, optic neuritis, Uhthoff's phenomenon, rehabilitation

## Abstract

125 years have passed since Wilhelm Uhthoff reported the symptoms he observed after an increased body temperature from physical exertion. Those symptoms, which might have led to the transient impairment of vision in patients with Multiple Sclerosis and also observed in optic neuritis, were later named after him "Uhthoff's phenomenon". This has defined the strategy of rehabilitation procedures in Multiple Sclerosis for more than 100 years, restricting the use of thermal treatments and the possibility of aerobic exercises. The current state of knowledge concerning the Uhthoff's phenomenon and its influence on comprehensive rehabilitation in Multiple Sclerosis were presented in the current review report.

## Introduction

In 1890, Professor Wilhelm Uhthoff - a founder of neuro-ophthalmology - described the phenomenon of transitory visual disturbance in patients with MS occurring after physical exercise and an increase in body temperature, e.g. after a hot bath. A lot of contradictory information on this topic has appeared since then both in neurology textbooks and over the Internet [**1**,**2**,**3**,**4**]. In 1961, this phenomenon was given his surname, Uhthoff's Phenomenon (UP), by G. Ricklefs. In four out of 100 MS patients, Uhthoff observed the appearance of reversible optic symptoms induced by an increase in body temperature, "marked deterioration of visual acuity during physical exercise and exhausting" [**5**]. 

In 1904, Uhthoff explained both the physical signs and etiologies of this phenomenon as symptoms which speak for a primary seat of the disturbance in the optic nerve [**6**,**7**].

### Uhthoff`s Phenomenon and Multiple Sclerosis- clinical manifestations and pathophysiology

The phenomenon described in 1890 by Uhthoff has been dictating the strategy for the physiotherapeutic procedure in MS for over 100 years. This led to a conservative approach limiting the application of heat forms of treatment and physical exertion during exercises, thereby diminishing the effectiveness of rehabilitation [**8**,**9**,**10**]. MS patients are too often prevented from doing hydrotherapy in water of a temperature exceeding 30°C and from any forms of treatment with high frequency waves (short-wave diatherapy) or paraffin applications. Often they are not allowed to use a sauna or hot showers. It is often feared that excessive physical exertion may provoke a new attack of the disease. It was recommended that patients should stay at home if the outside temperature exceeded 30°C, to use sunglasses, and to avoid tanning. Until 1983 "hot bath test" was used in the diagnosis of MS by obtaining increased body temperatures through the exposure of the organism body to infrared radiation or bathing in water of a temperature from 41.1 to 43.3°C for 10 to 15 minutes [**11**].

Lepore (1994) claimed that other factors besides heat might release UP: hot meals, menstruation, and even the smoking of cigarettes and mental stress [**12**]. Kohlmeier at al. described the case of the death of a forty-seven year old black MS patient in whom one of the causes of death was considered the heating of the organism body to a temperature of 40.94°C during a hot bath [**13**].

Saul et al. described the effect of hyperthermia (HT) on the central conduction by alterations in the pattern visual evoked potentials (PVEPs) in optic nerves in 10 normal subjects and in 6 patients with demyelinating optic neuropathy before and during HT. A mean rise in temperature of 2.5 degrees C in normal subjects resulted in a decrease of 6.1 ms (p < 0.0001) in the second positive peak (P2) latency and a slight decline of 1.16 muV (p < 0.009) in the P2 amplitude. These results were compared to those obtained from six patients with multiple sclerosis. These patients had a history of monocular optic neuritis; two patients had bilateral optic neuritis, and one patient did not have an involvement of the optic nerve. The average temperature elevations during PVEPs were 1.60 degrees C. PVEPs among these patients, showing a decrease in the mean P2 latencies, except for patients with multiple sclerosis, who showed an increase with 60 min in the latency, checking size in the left eyes. There was a consistent decline in P2 amplitudes. Loss of amplitude was greater among the six optic nerves of those patients with transient, mild losses in visual acuity during HT. The reductions in P2 amplitude were best explained by partial or complete conduction block. These changes in conduction time and amplitude during HT provide a neurophysiologic correlation to the well-known sensitivity of demyelinated optic nerves to elevated temperatures. They are also relevant for the monitoring of central pathways in the operative or intensive care setting. The demonstrated reversible loss of amplitudes also gives promise to therapeutic manipulation of impaired pathways by impeding the loss of current from denuded nerve fibers [**14**].

Triggered by the immune activation of the MS condition and possibly by heat, the innate immune response causes an up-regulation of the central nervous system (CNS) cytokine production. Central actions of proinflammatory cytokines, in particular IL-1, are pivotal for the induction of fatigue. It may be defined as a lack of energy or a feeling of exhaustion arising unconnected to depression or muscle weakness. Fatigue may occur in as many as 2/ 3rds of MS patients as one of the three main symptoms, and, in the opinion of patients, it may constitute the most troublesome symptoms of the disease. The independence of fatigue from the degree of motor disability and depression has been recently confirmed by American and German research [**15**,**16**,**17**,**18**,**19**].

A conservative approach in relation to the rehabilitation of MS was mainly held until the end of the twentieth century – avoiding the physical exertion as a factor that might trigger a new attack of the disease. However, several reports pointing to the effectiveness of aerobic exercise in MS have been published in recent years, fulfilling the requirements of Evidence-Based Medicine (EBM). It has been shown that by maintaining strict proper inclusion criteria for the qualification of the aerobic exercises, one may obtain improvement in the physical fitness of patients while not placing them at risk of a new relapse [[**20**,**21**,**22**,**23**,**24**]. However, according to Romberg et al. such exercise must last for at least a few weeks [**20**,**21**,**22**,**23**,**24**,**25**].

The UP is most often associated to optic neuritis (ON) occurring without symptoms, in several patients, even years before the manifestation of MS. The visual deterioration, usually unilateral, worsening over the course of a few days is characteristic, sometimes with pain in the eye and visual disturbances during ocular movements. In some cases, there may be atrophy of the affected optic nerve within 3-4. However, after 1-2 weeks visual acuity usually improves or returns to normal [**26**,**27**,**28**,**29**].

### Physiopathological explanations of Uhthoff’s phenomenon

A first attempt at a quantitative evaluation of the UP amongst 20 MS patients was conducted by Humm et al. They used motor evoked potentials to evaluate conduction velocity. Raising temperature led to the slowing of the conduction velocity in motor fibers (Central Motor Conduction Time – CMCT) - p=0.037 and a decrease in the speed of walking (p=0.0002) [**30**].

In 2001, Peterson described an interesting case challenging the cautions means sometimes applied in MS rehabilitation: a thirty three-year old woman with tetraparesis had been ill for three years and was admitted for comprehensive rehabilitation nine days after a relapse. Exercises in water of a temperature of 34.44°C were performed twice a week for 45 minutes, from the second week, and, after 6 weeks, significant improvements in independence, mobility, and muscle strength were documented without any deterioration in the neurological state or any fatigue [**31**].

Leigh and Serra stated in the Editorial that the study of Uhthoff’s phenomenon in patients has required considerable ingenuity because the measurement of an observed behavior (e.g., intention tremor) is often indirect, and because electrophysiological studies must take into account the complexities of the skeletal motor system (e.g., by using collision techniques) [**19**].

Based on the observation of the behavior of 8 patients with MS, Davis et al. claimed that an increase in the body temperature of 0.8°C results reduces the speed of adduction of the eye balls [**32**], by reversibly reducing the conduction velocity of the nerve fibers, and, it is assumed, that the diminishing of the body temperature through cooling may result in the reversal of such unfavorable symptoms. Frohman et al. demonstrated in a recent report that the adduction velocity in MS-related INO, as measured by infrared eye movement recording techniques, is further reduced by a systematic increase in core body temperature (utilizing tube-lined water infusion suits in conjunction with an ingestible temperature probe and transabdominal telemetry) and reversed to baseline with active cooling [**33**].

Davis et al. divided the current understanding of thermoregulatory dysfunction in MS into five problems: 1) heat sensitivity; 2) central regulation of body temperature; 3) thermo-regulatory effector responses; 4) heat-induced fatigue; and 5) countermeasures to improve or maintain function during thermal stress. According to their review article, an estimated 60–80% of the MS patients experience temporary worsens the clinical signs and neurological symptoms with heat exposure [**34**].

Fromont et al. reported four cases of patients presenting isolated UP preceding multiple sclerosis by several years. These four patients presented transient neurological symptoms induced by intensive sporting activity for 1 to 6 years before a diagnosis of MS could be established. These symptoms were often visual but sometimes also motor or sensory. All symptoms appeared after 15 to 30 minutes of intense physical exercise (bike, running or handball) and disappeared after a few minutes to one-hour rest with full recovery to baseline. In these cases, UP was explained by a conduction block due to axonal demyelination leading to the reorganization of sodium channels or by the release of soluble blocking substances (e.g. nitric oxide or cytokines). The “safety factor” appeared to be highly sensitive to temperature. The authors concluded that without being specific, this symptom was strongly suggestive of MS [**35**].

In a recent report, Dodd et al. proved that progressive resistance training (PRT) does not improve walking but may improve muscle performance, quality of life, and fatigue in adults with MS. In a randomized controlled trial, people with relapsing-remitting MS were randomly allocated to either a PRT program for lower limb muscles twice a week for 10 weeks (n = 36), or usual care plus an attention and social program once a week for 10 weeks (n = 35). The outcomes were recorded at baseline, week 10, and week 22. At 10 weeks, no differences were detected in walking performance. However, compared to the control group, PRT led to an increased leg press strength (16.8%, SD 4.5), increased reverse leg press strength (29.8%, SD 12.7), and increased muscle endurance of the reverse leg press (38.7%, SD 32.8). Improvements in favor of PRT were also found for physical fatigue (Mean difference -3.9 units, 95%CI -6.6 to -1.3), and the physical health domain of quality of life (Mean difference 1.5 units, 95%CI 0.1 to 2.9). On week 22, almost no between-group differences remained. In conclusion: PRT was a relatively safe intervention that could have short-term effects on reducing physical fatigue, increasing muscle endurance and could lead to small improvements in muscle strength and quality of life in people with relapsing-remitting MS [**36**].

Fraser et al. assessed phenotypes of UP. A one-page questionnaire was sent to 80 consecutive optic neuritis (ON) patients seen in a tertiary neuro-ophthalmology clinic. Of the 48 responders to the questionnaire, 52% reported experiencing UP, with a follow-up range of 1 to 20 years. Only 16% showed a complete resolution of UP within 8 weeks. Of the MS patients with UP, 88% experienced non-visual heat-related phenomena compared with 30% without UP. The authors concluded that the presence of UP may have a more general phenotypic significance. If full recovery from UP has not occurred within the first 2 months after onset of ON, recovery is uncommon and may therefore serve as a surrogate marker of remyelination in the future drug trials [**37**].

Guthrie and Nelson stated that over 80% of the MS patients develop a panoply of neurological signs during hyperthermia, 60% of which are “new” to that patient. The literature contains a number of unexplained paradoxical responses of MS patients during induced hyperthermia. These challenge the current hypothesis that, in MS, hyperthermia induces a heat-linked neuro-blockade of partially demyelinated axons [**38**].

Sa recently stated that the physiopathological basis for the Uhthoff’s phenomenon has been attributed to demyelination and consequent reduction in axonal cross-sectional area, thereby decreasing the conduction velocity, and to loss of internodal conduction, with a predisposition to conduction slowing and block. The warming might change the electrical properties of the demyelinated axon and block of conduction ensues thorough an increase in the rate of recovery processes (potassium channel activation and sodium channel inactivation), which surpass the action potential, generating processes (sodium channel activation) [**39**].

Park et al. (2014) compared the incidence and clinical features of UP in Japanese patients with neuromyelitis optica (NMO) and those with MS. This was the first report of the frequency of UP in Asian MS patients. They asked 135 consecutive patients with MS and an NMO-related disorder (NMOrd) whether they experienced worse neurological symptoms after an increase in body temperature. They included patients with typical UP symptoms: weakness, sensory symptoms (hypesthesia, pain, and numbness), and visual symptoms (blurred vision and visual loss). Responses were obtained from 54 MS and 37 NMOrd patients. Uhthoff’s phenomenon was observed in 26 MS (48.1%) and 20 NMOrd patients (54.1%). Motor and sensory symptoms were more frequent than visual symptoms in both diseases. The incidence of UP occurred similar in MS and NMOrd [**40**].

Muto et al. (2015) investigated the frequencies of symptoms and signs, previously regarded as characteristic of MS, such as Lhermitte’s sign, UP and painful tonic seizure in 128 Japanese MS patients, and in 48 patients with Neuromyelitis Optica – NMO (NMO-plus patients n = 30 or partial NMO n = 18), which is another inflammatory disease of the central nervous system (most of the opticospinal form of MS is thought to be NMO).

Univariate analyses revealed that tonic seizures, Lhermitte’s sign, persistent pain, fatigue and girdle sensation were more frequent in NMO-plus patients than in MS patients. Multivariate logistic regression analysis showed that paroxysmal itching, UP, Lhermitte’s sign, and girdle sensation were more characteristic of NMO-plus than of MS. The authors’ conclusions were the following: several classical MS symptoms and signs are more frequent in NMO patients than in MS patients, which may be caused by the differences in the severity of inflammation, and localization and extensiveness of demyelinated lesions [**41**].

The elevated body temperature was recently reported for the first time in 2014 in patients with relapsing-remitting multiple sclerosis (RRMS). In addition, warmer body temperature was associated with worse fatigue. These findings, which are highly novel, may indicate a novel pathophysiology for UP. Leavitt et al. investigated the body temperature and its association to fatigue in an Italian sample of 44 RRMS patients and 44 healthy controls. They found elevated body temperature in the RRMS sample (mean ± SD 37.06 ± .26°C) relative to healthy controls (mean ± SD 36.89 ± 0.31°C), t(86) = -2.80, P = 0.003). A warmer body temperature was associated with worse fatigue, thereby supporting the notion of endogenous temperature elevations in patients with RRMS as a novel pathophysiological factor underlying fatigue. Those findings highlighted a paradigm shifting the effect of heat in RRMS, from exogenous (i.e., UP) to endogenous. Although randomized controlled trials of cooling treatments (i.e., aspirin, cooling garments) to reduce fatigue in RRMS have been successful, the consideration of endogenously elevated body temperature as the underlying target will enhance the development of novel treatments [**42**].

In a cross-sectional study with 50 RRMS patients, Sumowski and Leavitt matched 40 healthy controls and 22 patients with Secondary Progressive Multiple Sclerosis (SPMS) and confirmed that the body temperature is elevated and linked to fatigue in RRMS, even without heat exposure. There was a large effect of group (P<.001, ηp(2)=.132) whereby body temperature was higher in patients with RRMS (37.04°±.27°C) relative to healthy controls (36.83°±.33°C; P=.009) and patients with SPMS (36.75°±.39°C; P=.001). Warmer body temperature in patients with RRMS was associated with worse general fatigue (FSS; rp=.315, P=.028) [**43**].

## Discussion and key messages

Research carried out in recent years has shed more light on the Uhthoff’s phenomenon. However, the influence of heating on MS patients is still not clear-cut. In a Japanese study by Park et al., the most common cause of UP was bathing, which might be attributable to the different lifestyles of Japanese people. In Japan, “Taking a bath” usually means soaking in the bathwater. According to those authors, the worsening of the neurological symptoms after an increase in body temperature can be reduced by the oral administration of 4-aminopyridine, which inhibits potassium channel activation, thereby prolonging the action potential, which can restore nerve conduction in demyelinated nerve fibers. Recent clinical trials of dalfampridine detected an improvement in the gait disturbance of MS patients, thus it may have beneficial effects on the neurological symptoms in NMOrd patients [**42**].

Leavitt et al. acknowledged that a multitude of factors such as circadian rhythms (time of day) and menstrual cycles influence body temperature. That is, body temperature fluctuates within persons throughout the day, with lower body temperatures in the early morning and late evening/ night [**43**].

Up to now, few questions still remained to be answered;

1. Should hot hydrotherapy in water of a temperature exceeding 30°C, high frequency waves (short-wave diatherapy) and paraffin applications be restricted for all MS patients or just for those suffering from optic neuritis (ON)?

2. If the patient had ON in passed time but now has no symptoms of it could we order heat therapy? 

3. If yes, which should be the time distance from ON to order heat therapy? 
